# Incidence and risk factors for postoperative posterior column fractures following periacetabular osteotomy

**DOI:** 10.1093/jhps/hnaf059

**Published:** 2025-09-18

**Authors:** Yasuhiko Kokubu, Shinya Kawahara, Goro Motomura, Satoshi Hamai, Satoshi Ikemura, Masanori Fujii, Taishi Sato, Ryosuke Yamaguchi, Daisuke Hara, Takeshi Utsunomiya, Kenji Kitamura, Yasuharu Nakashima

**Affiliations:** Department of Orthopaedic Surgery, Graduate School of Medical Sciences, Kyushu University, 3-1-1 Maidashi, Higashi-ku, Fukuoka City, Fukuoka Prefecture 812-8582, Japan; Department of Orthopaedic Surgery, Graduate School of Medical Sciences, Kyushu University, 3-1-1 Maidashi, Higashi-ku, Fukuoka City, Fukuoka Prefecture 812-8582, Japan; Department of Orthopaedic Surgery, Graduate School of Medical Sciences, Kyushu University, 3-1-1 Maidashi, Higashi-ku, Fukuoka City, Fukuoka Prefecture 812-8582, Japan; Department of Orthopaedic Surgery, Graduate School of Medical Sciences, Kyushu University, 3-1-1 Maidashi, Higashi-ku, Fukuoka City, Fukuoka Prefecture 812-8582, Japan; Department of Orthopaedic Surgery, Graduate School of Medical Sciences, Kyushu University, 3-1-1 Maidashi, Higashi-ku, Fukuoka City, Fukuoka Prefecture 812-8582, Japan; Department of Orthopaedic Surgery, Graduate School of Medical Sciences, Kyushu University, 3-1-1 Maidashi, Higashi-ku, Fukuoka City, Fukuoka Prefecture 812-8582, Japan; Department of Orthopaedic Surgery, Faculty of Medicine, Saga University, 5-1-1 Nabeshima, Saga City, Saga Prefecture 849-8501, Japan; Department of Orthopaedic Surgery, Graduate School of Medical Sciences, Kyushu University, 3-1-1 Maidashi, Higashi-ku, Fukuoka City, Fukuoka Prefecture 812-8582, Japan; Department of Orthopaedic Surgery, Graduate School of Medical Sciences, Kyushu University, 3-1-1 Maidashi, Higashi-ku, Fukuoka City, Fukuoka Prefecture 812-8582, Japan; Department of Orthopaedic Surgery, Graduate School of Medical Sciences, Kyushu University, 3-1-1 Maidashi, Higashi-ku, Fukuoka City, Fukuoka Prefecture 812-8582, Japan; Department of Orthopaedic Surgery, Graduate School of Medical Sciences, Kyushu University, 3-1-1 Maidashi, Higashi-ku, Fukuoka City, Fukuoka Prefecture 812-8582, Japan; Department of Orthopaedic Surgery, Graduate School of Medical Sciences, Kyushu University, 3-1-1 Maidashi, Higashi-ku, Fukuoka City, Fukuoka Prefecture 812-8582, Japan; Department of Orthopaedic Surgery, Graduate School of Medical Sciences, Kyushu University, 3-1-1 Maidashi, Higashi-ku, Fukuoka City, Fukuoka Prefecture 812-8582, Japan

## Abstract

Periacetabular osteotomy (PAO) is a widely used surgical technique for symptomatic developmental dysplasia of the hip (DDH). However, the incidence of posterior column fractures after PAO and the risk factors are not adequately investigated. Identification rates have been improved by using oblique radiographs, but few reports have examined the incidence of posterior column fractures using early postoperative computed tomography (CT) scans. This study aimed to investigate the incidence and risk factors for postoperative posterior column fracture following PAO using early postoperative CT scans. This study included 80 hips from patients who underwent PAO for symptomatic DDH. Radiographic parameters such as lateral center-edge angle (LCEA), anterior center-edge angle (ACEA), acetabular roof obliquity (ARO), and the thickness of the posterior column before and after surgery were evaluated using preoperative and early postoperative (within 1 month) CT images. Statistical analyses were conducted to identify risk factors associated with postoperative posterior column fracture. Postoperative CT scans revealed that postoperative posterior column fracture occurred in 42% of cases. A preserved posterior column thickness of <13 mm was identified as a significant risk factor for postoperative posterior column fracture. In conclusion, Posterior column fractures are a relatively common complication following PAO, with a higher incidence than previously reported. A preserved posterior column thickness of <13 mm is a critical risk factor for postoperative posterior column fracture. These results suggest that early weight-bearing should be approached with caution and that accurate osteotomy techniques using computer-assisted surgery are needed.

## INTRODUCTION

Developmental dysplasia of the hip (DDH) is a common cause of secondary osteoarthritis (OA) [1–3]. Periacetabular osteotomy (PAO) was developed to correct acetabular coverage of the femoral head to delay or prevent the progression of secondary OA. This surgical procedure is commonly used to treat symptomatic hip dysplasia in young adults, and several studies have reported favorable long-term outcomes [2, 4]. However, the surgical procedure for PAO is challenging because it requires a sufficient understanding of the pelvic anatomy and skillful surgical techniques. Hence, various complications such as intra-articular perforation, thin acetabular fragments, and posterior column fractures have often been reported [5–8].

Among these complications, posterior column fractures following PAO are difficult to identify using anteroposterior radiographs [9]. However, recent reports have indicated that the sensitivity of detecting posterior column fractures following PAO has significantly improved using oblique radiographs, including false profile view [5]. Several studies have reported that computed tomography (CT) is more sensitive than radiography in detecting fractures [10, 11], and postoperative posterior column fractures may be detected with even higher sensitivity on CT scans. However, few reports have examined the incidence of posterior column fractures on CT. Moreover, the risk factors for postoperative posterior column fractures based on patient factors or anatomical characteristics have not been reported. The posterior column of the pelvis, which is the load-bearing pathway, becomes thin after PAO. This study aimed to identify the incidence and risk factors of posterior column fractures following PAO using early postoperative CT scans. We hypothesized that a preserved thin posterior column is a critical risk factor for postoperative posterior column fracture.

## METHODS

This study included patients who had undergone PAO for symptomatic DDH between November 2018 and March 2024 (Fig. 1). Patients who had undergone PAO using a navigation system in a different study were also excluded. These patients were selected based on a randomized controlled trial, and there was no patient selection bias. The indication for PAO was radiological evidence of dysplasia with a lateral center-edge angle (LCEA) <25° in patients who experienced hip pain interfering with daily activities [2, 3, 6, 12].

**Figure 1:**
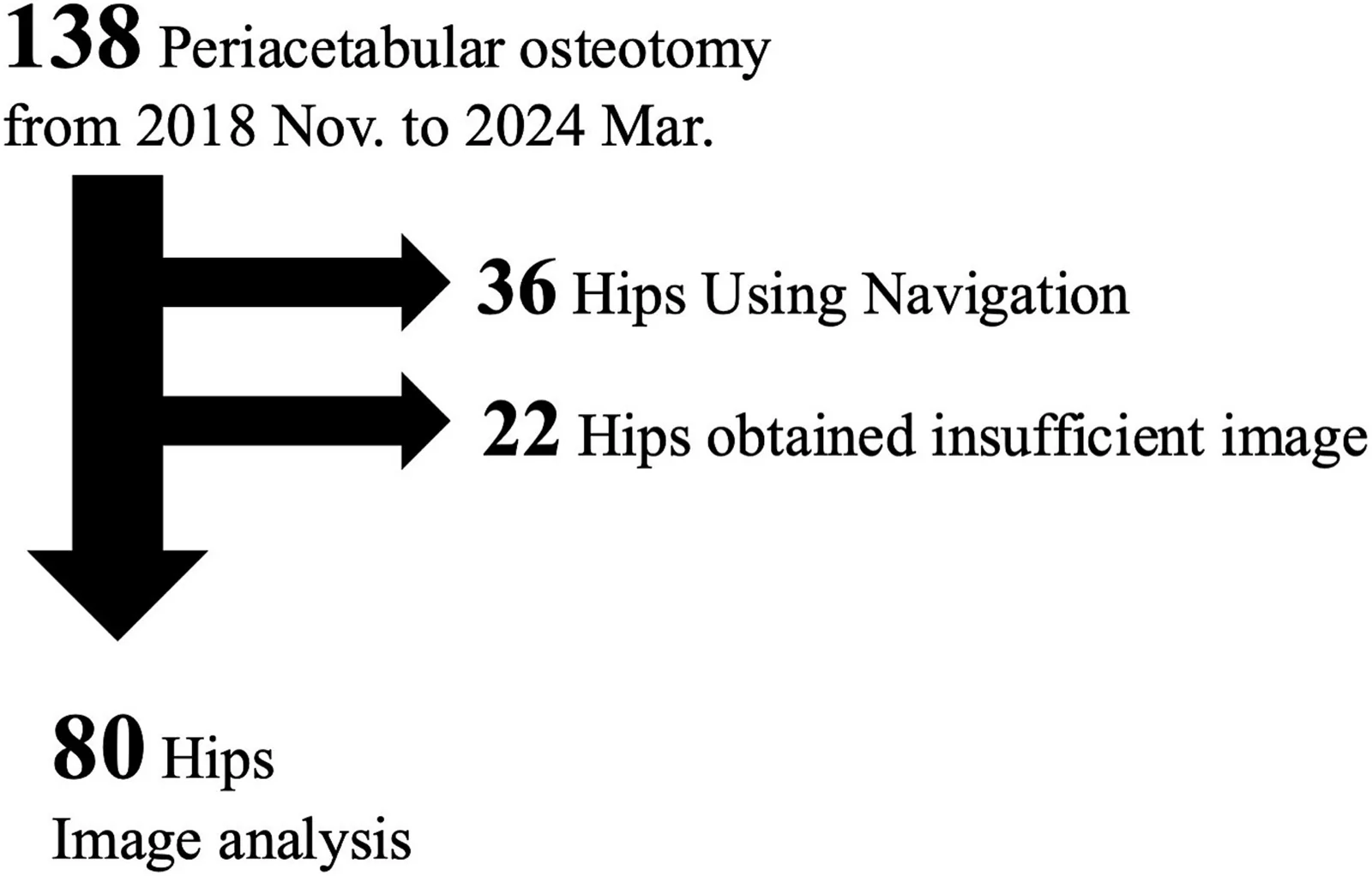
Strengthening the reporting of observational studies in epidemiology diagram for the inclusion process.

All PAO procedures were performed at the same institution by nine experienced hip surgeons using consistent techniques and intraoperative fluoroscopic guidance. The surgery was performed based on the modified transposition osteotomy of the acetabulum, as reported by Nakashima *et al.* [13], which is a modification of the traditional lateral approach described by Nishio [14]. This modified method was performed in the same manner as the conventional method without cutting the greater trochanter or inducing abductor-iliac crest detachment. Briefly, surgery was performed with the patient in the lateral decubitus position using an Ollier skin incision. The short external rotator muscles were detached to expose the posterior aspect of the joint capsule, ilium, and ischium. The gluteus medius and tensor fasciae latae were partially detached anteriorly. Instead of performing a trochanteric osteotomy, the osteotomy site was exposed by retracting the abductor muscles using an abductor retractor to avoid iliac crest detachment. The joint capsule was circumferentially exposed, and spherical osteotomy was performed using a curved osteotome, starting 20 mm proximally from the superior acetabular margin and extending through the midpoint between the superior rim of the acetabular fossa and the ischial spine. Pubic osteotomy was performed laterally to the pubic tubercle. The acetabular fragment was then adducted to achieve a horizontal weight-bearing surface and rotated anteriorly to correct the anterior coverage deficiency. The fragment was fixed to the pelvis using 2–3 cortical screws (diameter, 4.5 mm) (Fig. 2). Postoperatively, patients were allowed wheelchair transfer from the first day, started hip range of motion exercises from the second day, and began partial weight-bearing from two weeks postoperatively, progressing to full weight-bearing by 8–10 weeks. In six cases, the conventional surgical method with greater trochanter osteotomy was performed to ensure a sufficient surgical field.

**Figure 2:**
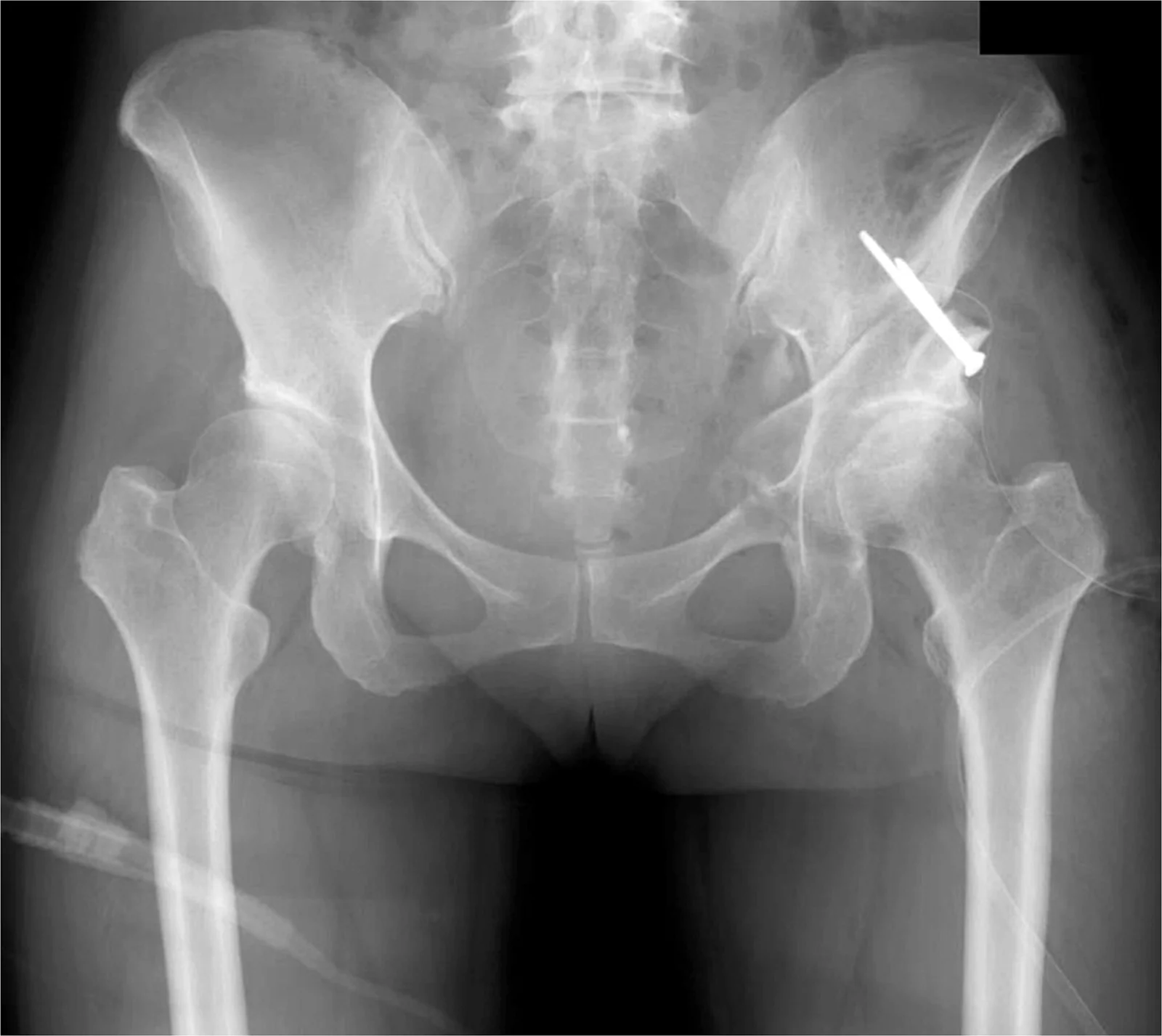
Postoperative radiograph after periacetabular osteotomy showing sufficient correction with a horizontal weight-bearing acetabulum.

Postoperative follow-up included pelvic radiographs in the anteroposterior view for all patients (weekly until 3 weeks postoperatively, and every 2–3 weeks thereafter according to the progression of weight-bearing status) and routine CT scans within one month postoperatively to evaluate the reorientation of the acetabulum. Based on these selection criteria, the following were excluded: 36 hips that underwent PAO using a navigation system; 22 hips that could not undergo CT scans during the postoperative hospitalization period; and hips that were transferred to a rehabilitation hospital. After assessing for eligibility, 80 hips were enrolled in this study (Fig. 1). This retrospective study was approved by the Institutional Review Board for clinical research (No. 2023–107) and conducted in accordance with the Declaration of Helsinki. Written informed consent was obtained from all patients before the study.

### Image analysis

Preoperative and postoperative transverse CT scans (Aquilion ONE; Canon Medical Systems Corporation, Tochigi, Japan) of the whole pelvis and femur (from the top of the iliac crest to the distal femur) were obtained from all patients with a 512 × 512 image matrix, a 0.35 × 0.35 pixel dim, and a 1-mm thickness. Patients were placed supine on a scanning table and instructed to naturally extend their affected hip and knee joints. CT images were acquired using the digital imaging and communications in medicine (DICOM) format data from a CT system server. The DICOM datasets were imported into a 3D planning software (Zed View; Lexi Co., Ltd., Tokyo, Japan), and radiological parameters were evaluated using CT images. LCEA and acetabular roof obliquity (ARO) were assessed in the coronal plane passing through the center of the femoral head, and the anterior center-edge angle (ACEA) was evaluated in the sagittal plane passing through the center of the femoral head [3, 15]. To evaluate the amount of correction, changes in radiographic parameters (ΔLCEA, ΔACEA, and ΔARO) were calculated by subtracting preoperative values from postoperative values. Posterior column fractures were investigated using postoperative CT, with fractures defined as cortical discontinuities visible on coronal, sagittal, or axial images at the level of the posterior column. The thickness of the posterior column before and after surgery was measured using 3D models reconstructed from the CT images, with measurements performed at the thinnest part of the posterior column (Fig. 3).

**Figure 3:**
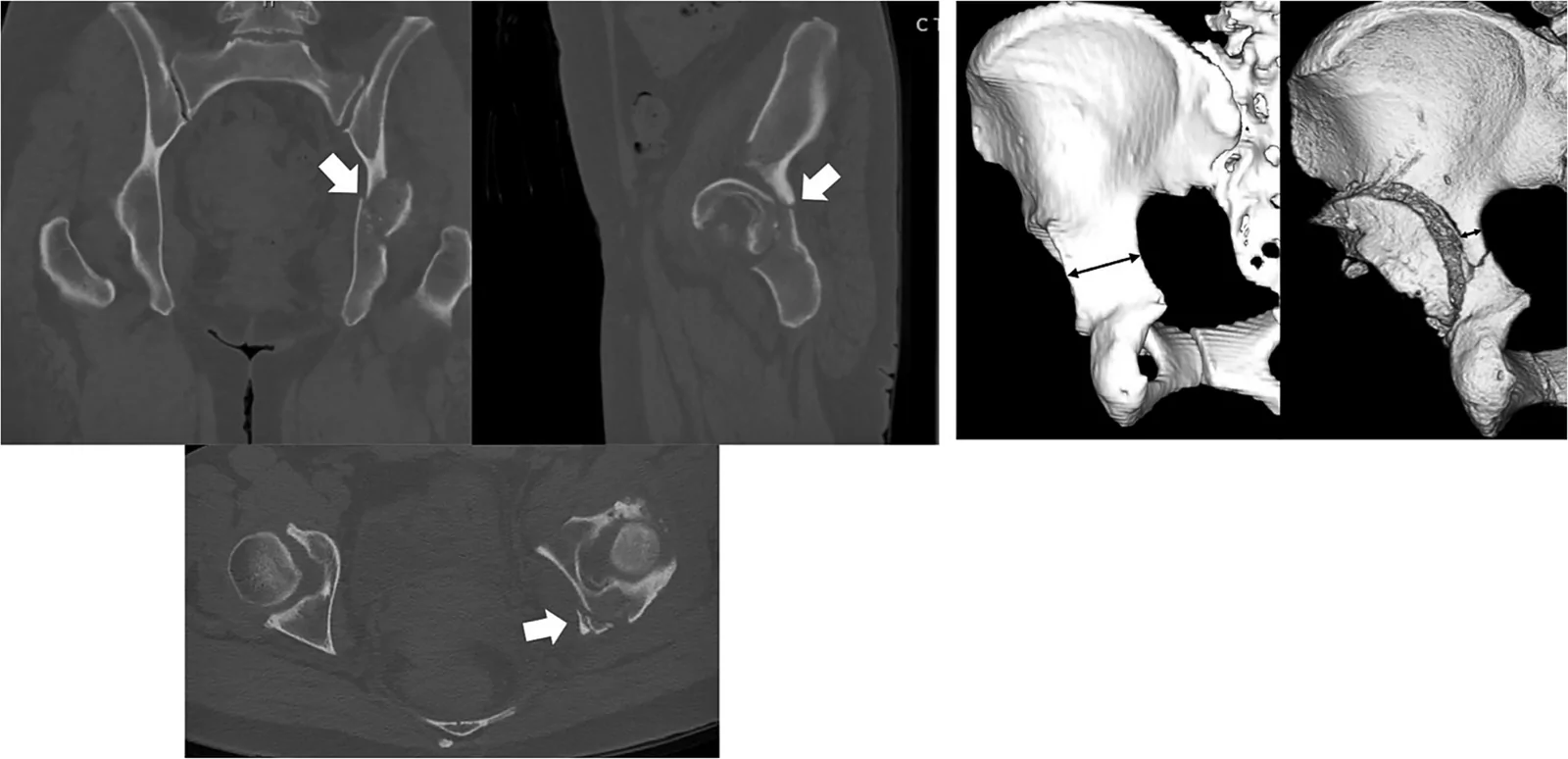
Posterior column fractures were identified with reference to the coronal, sagittal, and axial planes of the postoperative CT images. The thickness of the posterior column before and after surgery was measured on 3D models reconstructed from CT images.

### Statistical analysis

The patients were divided into two groups based on the presence of postoperative posterior column fractures. Categorical variables were compared using Fisher’s exact test and the chi-squared test, whereas continuous variables were compared using the Mann–Whitney U test and t-test, depending on the normality (Shapiro–Wilk test). Statistical significance was set at *P*< .05. Receiver operating characteristic (ROC) curves were generated to set reference values for independent factors associated with postoperative posterior column fractures. Continuous variables are presented as means and standard deviations (SD). Statistical analyses were performed using JMP (statistical analysis software; version 15.0; SAS Institute, Cary, NC, USA).

## RESULTS

In the entire cohort, postoperative LCEA, ACEA, and ARO showed good improvement of 40.4 ± 9.9°, 49.7 ± 11.5°, and − 5.6 ± 8.8°, respectively, resulting in sufficient femoral head coverage (Table 1). Postoperative CT scans revealed postoperative posterior column fractures in 34 patients (42%). Among the 34 patients, two patients also had associated inferior ramus fractures, which led to delayed full weight-bearing. When the parameters between the fracture and non-fracture groups were compared, the preserved posterior column was thinner in the fracture group (Table 2). ROC analysis indicated that a preserved posterior column thickness of <13 mm postoperatively was a risk factor for postoperative posterior column fractures (Fig. 4).

**Table 1:** Descriptive data and radiographic data.

Parameters	*n* = 80
Age at surgery (year)	38.8 ± 10.7
Sex, Male, *n* (%)	7 (9)
Sex, Female, *n* (%)	73 (91)
Height (cm)	159.2 ± 6.8
Weight (kg)	59.6 ± 11.8
BMI (kg/m^2^)	23.5 ± 4.4
Preoperative LCEA (°)	13.3 ± 8.2
Preoperative ACEA (°)	39.2 ± 14.6
Preoperative ARO (°)	19.4 ± 7.6
Preopeative posterior column thickness (mm)	35.2 ± 3.5
Postoperative LCEA (°)	40.4 ± 9.9
Postoperative ACEA (°)	49.7 ± 11.5
Postoperative ARO (°)	−5.6 ± 8.8
Postoperative posterior column thickness (mm)	15.1 ± 4.0
Postoperative Posterior column fracture, *n* (%)	34 (42)

Note: Values are given as the mean and SD.

Abbreviations: PCF, posterior column fracture. BMI, body mass index. LCEA, lateral center-edge angle. ACEA, anterior center-edge angle. ARO, acetabular roof obliquity.

**Table 2:** Descriptive data and radiographic data.

Parameters	PCF (*n* = 34)	No PCF (*n* = 46)	*P*-value
Age at surgery (year)	39.3 ± 10.6	38.5 ± 10.8	.7509
Sex, Male, *n* (%)	3 (9)	4 (9)	1.0
Sex, Female, *n* (%)	31 (91)	42 (91)	1.0
Height (cm)	158.2 ± 6.2	159.9 ± 7.2	.2685
Weight (kg)	59.5 ± 12.9	59.7 ± 11.1	.9466
BMI (kg/m^2^)	23.8 ± 4.7	23.54 ± 4.2	.7002
Preoperative LCEA (°)	12.9 ± 10.0	13.5 ± 6.6	.7697
Preoperative ACEA (°)	39.6 ± 16.9	39.0 ± 12.9	.8614
Preoperative ARO (°)	18.9 ± 8.9	19.7 ± 7.1	.6530
Postoperative LCEA (°)	40.6 ± 11.3	40.2 ± 8.8	.8551
Postoperative ACEA (°)	49.8 ± 11.6	49.5 ± 11.5	.9223
Postoperative ARO (°)	−5.2 ± 10.0	−5.8 ± 7.9	.7845
ΔLCEA (°)	27.7 ± 9.0	26.7 ± 7.4	.9534
ΔACEA (°)	10.2 ± 14.4	10.6 ± 11.9	.6579
ΔARO (°)	−23.8 ± 9.8	−25.2 ± 8.1	.4714
Preoperative posterior column thickness (mm)	35.8 ± 3.6	34.8 ± 3.4	.2262
Postoperative posterior column thickness (mm)	12.6 ± 3.2	16.7 ± 3.7	**<.0001**

Note: Values are given as the mean and SD.

Abbreviations: PCF, posterior column fracture. BMI, body mass index. LCEA, lateral center-edge angle. ACEA, anterior center-edge angle. ARO, acetabular roof obliquity. Bold values indicate that a significant difference between the two groups was observed only in postoperative posterior column thickness.

**Figure 4:**
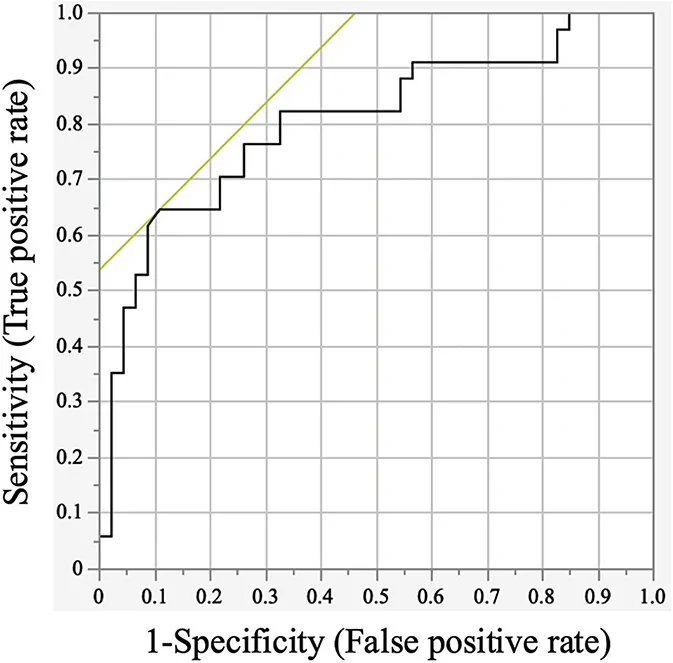
The receiver operating characteristic curve for postoperative posterior column fractures following periacetabular osteotomy. The cut-off value of the preserved posterior column thickness was 13 mm (sensitivity 65%, specificity 89%, area under the curve 0.80).

## DISCUSSION

In this study, posterior column fracture was observed in 42% of cases following PAO, and a preserved posterior column thickness of <13 mm was identified as a risk factor.

Postoperative posterior column fracture is a complication reported in 3% of cases following PAO [9]. These lower reported incidence rates were based on anteroposterior radiographs and intraoperative fluoroscopic investigations. However, Morris *et al.* reported a 17% higher incidence of posterior column fractures with postoperative follow-up using oblique radiographic images such as false-profile radiographs [5]. They noted that the sensitivity of detecting posterior column fractures was low on anteroposterior radiographs and fluoroscopy, which might have led to the under-recognition of posterior column fractures as a major complication after PAO. The strength of this study is the routine use of CT scans, which have a higher sensitivity for detecting fractures in the early postoperative period. The study revealed a higher incidence of posterior column fractures than previously reported. Thus, we found that posterior column fractures following PAO occurred at a higher rate of 38%. However, the rehabilitation protocol for patients with PAO remains controversial [16, 17]. Although an accelerated rehabilitation protocol seems beneficial for early muscle recovery [16], the results of this study suggest that early weight-bearing should be approached with caution.

The risk factor for a preserved posterior column thickness of <13 mm in posterior column fractures is a novel finding. Considering that the posterior column is a load-bearing pathway through the pelvic ring and there is increased loading stress on the posterior column after PAO [16, 18], it is reasonable to assume that a thinner preserved posterior column increases the risk of fracture. The preoperative thickness of the posterior column was 27–42 mm in the entire cohort, with no significant differences between the fracture and non-fracture groups. A thickness of 13 mm was sufficient for preservation if the chisel could pass through the midpoint of the posterior column, as planned. The osteotomy process requires extreme precision; therefore, the use of computer-assisted surgery, such as navigation systems, may improve the accuracy [8, 19].

This study had several limitations. First, this study was limited to a single surgical technique using a lateral approach and spherical osteotomy. Although other approaches (such as Bernese PAO) may have different mechanical impacts due to different surgical procedures, the importance of preserving the thickness of the posterior column is considered to be applicable to various techniques. Moreover, we did not evaluate bony contact or healing at the superior pubic ramus osteotomy site, which may influence posterior load transmission. Prior studies on Bernese PAO have suggested that nonunion at the pubic ramus may be associated with increased risk of posterior column fractures [20]. Second, not all patients who underwent PAO were included in the study. Early postoperative CT scans were not obtained in all cases. In a general hospital with multiple departments, CT scans are performed on an emergency priority basis, using a limited number of scans. Consequently, in some cases, CT scans could not be performed during hospitalization but were performed after discharge. Thirty-six patients who had undergone PAO using a navigation system were excluded. However, these patients were selected based on randomized trials for another study currently under investigation. Therefore, a selection bias was minimal, as severe DDH cases were not selected for navigation. Further investigation is needed to assess the fracture risk with computer-assisted PAO. These cases were excluded from the study because of their potential impact on the fracture detection sensitivity, but there were no demographic differences between excluded and included patients (Appendix Table 1). Third, patients were generally rehabilitated with the same protocol of partial loading for a minimum of 8 weeks, but were not strictly monitored for the time to full weight-bearing. Furthermore, although this study focused mainly on radiological parameters, other factors such as bone density may also be risk factors for fractures. Finally, postoperative CT scans were routinely performed at our institution for accurate assessment of acetabular reorientation; however, we acknowledge that this may not be feasible in all clinical settings due to concerns about radiation exposure and cost.

## CONCLUSION

We evaluated early postoperative CT scans after PAO. Posterior column fractures occurred in 42% of cases and a preserved posterior column thickness of <13 mm was identified as a significant risk factor.

## Supplementary Material

Appendix_Table_1_hnaf059

## Data Availability

The datasets used and/or analyzed during the current study are available from the corresponding author on reasonable request.
