# Supplementary material for: Incidence and risk factors for postoperative posterior column fractures following periacetabular osteotomy
Source: J Hip Preserv Surg. 2025 Sep 18;13(3):213–8. doi: 10.1093/jhps/hnaf059 (PMC13653051; doi:10.1093/jhps/hnaf059)
Supplement: Supplementary file 1 [file appendix_table_1_hnaf059.docx]

**Appendix table 1**. Descriptive data and radiographic data

| Parameters | Included (n = 80) | Excluded (n=58) | p-value |
| --- | --- | --- | --- |
| Age at surgery (year) | 38.8 ± 10.7 | 38.6 ± 10.0 | 0.6754 |
| Sex, Male, n (%) | 7 (9) | 7 (12) | 0.5756 |
| Sex, Female, n (%) | 73 (91) | 51 (88) | 0.5756 |
| Height (cm) | 159.2 ± 6.8 | 158.0 ± 6.6 | 0.3488 |
| Weight (kg) | 59.6 ± 11.8 | 57.6 ± 8.9 | 0.1254 |
| BMI (kg/m^2^) | 23.5 ± 4.4 | 23.0 ± 3.4 | 0.2118 |

Note: Values are given as the mean and standard deviation.

Abbreviations: BMI, body mass index
